# Virus-specific RNA and Antibody from Convalescent-phase SARS Patients Discharged from Hospital

**DOI:** 10.3201/eid1010.040026

**Published:** 2004-10

**Authors:** Hoe Nam Leong, Kwai Peng Chan, Ali S. Khan, Lynette Oon, Su Yun Se-Thoe, Xin Lai Bai, Daniel Yeo, Yee Sin Leo, Brenda Ang, Thomas G. Ksiazek, Ai Ee Ling

**Affiliations:** * Tan Tock Seng Hospital, Singapore;; † Singapore General Hospital, Singapore;; ‡ Centers for Diseases Control and Prevention, Atlanta, Georgia, USA;; § Global Outbreak Alert and Response Network, Geneva, Switzerland

**Keywords:** SARS, convalescence, polymerase chain reaction, serology, ELISA, Indirect Immunofluorescence Assay, research

## Abstract

Severe acute respiratory syndrome (SARS) is caused by a novel coronavirus (SARS-CoV). In a longitudinal cross-sectional study, we determined the prevalence of virus in bodily excretions and time of seroconversion in discharged patients with SARS. Conjunctival, throat, stool, and urine specimens were collected weekly from 64 patients and tested for SARS-CoV RNA by real-time polymerase chain reaction; serum samples were collected weekly and tested for SARS-CoV antibody with indirect enzyme immunoassay and immunofluorescence assay. In total, 126 conjunctival, 124 throat swab, 116 stool, and 124 urine specimens were analyzed. Five patients had positive stool samples, collected in weeks 5–9. Two patients seroconverted in weeks 7 and 8; the others were seropositive at the first serum sample collection. In this study, 5 (7.8%) of 64 patients continued to shed viral RNA in stool samples only, for up to week 8 of illness. Most seroconversions occurred by week 6 of illness.

## Abstract 2

The prevalence of SARS-CoV in bodily excretions was determined.

Severe acute respiratory syndrome (SARS), a newly-defined condition identified in March 2003, is caused by the novel SARS-associated coronavirus (SARS-CoV). It is believed to have originated in Guangdong Province, China, with subsequent spread to other cities and countries. By August 15, 2003, a total of 30 countries had reported cases of SARS; 5 countries had local transmission. Approximately 8,500 cases were reported and at least 916 deaths (*1*). Singapore's index case-patient was a 22-year-old woman admitted to the hospital for atypical pneumonia on March 1, 2003; she had returned from a trip to Hong Kong on February 25. An additional 237 cases were identified and 33 deaths. The date of onset of illness of the last patient was May 5, 2003.

The role of body fluids in transmission and the potential for transmission during convalescence remain undefined. In Hong Kong, for example, faulty sewage works in a residential block were deemed to be the cause of a major outbreak in a residential estate (*2*). Excretion of the virus RNA in bodily fluids has also been reported during the convalescent phase (*3*), despite the patient's apparent clinical recovery. Detecting SARS-CoV in excreted bodily fluids would thus have substantial implications on infection control measures for recovered patients returning to the community.

The relationship of potential virus secretion to seroconversion is also undefined, as is the optimal time to test for seroconversion. Seroconversion has been suggested to occur at a mean of 20 days of illness (*4*). Recently available laboratory tests for detecting antibodies against SARS-CoV provide a means to answer these clinical questions.

A longitudinal cross-sectional study was designed to study the prevalence of the virus and the duration of viral shedding in bodily excretions (stool, urine, throat secretions, and conjunctival tears) by using nucleic acid amplification tests on samples from patients who had recently recovered from SARS. We also sought to determine the factors for viral shedding and the time of seroconversion in patients recovering from SARS.

## Methods

### Patients

Patients for the study were recruited at the Tan Tock Seng Hospital, Singapore, from April 30 to May 30, 2003. The government designated this hospital for all SARS-infected patients. A total of 233 of the 238 patients with reported probable cases of SARS in Singapore were cared for at this hospital. Approval for this study was obtained from the Ministry of Health, Singapore, and the local hospital ethics committee. Consent was obtained from all recruited patients.

Patients were identified from the existing patient registry maintained at the hospital. Selection criteria were a diagnosis of SARS based on current World Health Organization (WHO) recommendations (*5*) and being a discharged patient. Patients were recruited into the study anytime from the day of discharge up to 42 days after discharge. Discharge criteria were based on existing WHO recommendations (*6*). In our study, the endpoint of collection was arbitrarily set at 42 days after discharge. Given an estimated 14- to 21-day hospital stay for each patient, our study design would allow sampling up to days 56 to 63 of illness. During the initial study design, one report (*3*) showed viral RNA in stool samples from convalescent-phase patients up to day 25 of illness. Collection of specimens up to day 63 appeared sufficient. A dedicated team of four doctors collected weekly specimens from the throat and conjunctiva from each consenting patient. Fresh specimens of midstream urine, stool, and blood were collected concurrently in sterile specimen bottles.

### Sample Collection

A sterile swab was used to collect throat secretions from the fornices and back of the throat. A spatula was used to depress the tongue during the procedure. The swab was then immediately placed in a bottle containing Hank's viral transport medium. By using a sterile swab, the conjunctiva of one lower eyelid was swabbed with one brush. The swab was then rotated 180°, and the conjunctiva of the other lower eyelid was then swabbed similarly. The swab was immediately placed in a bottle containing Hank's viral transport medium. Midstream urine was collected in a sterile urine container. Stool specimens were collected with a spatula into a sterile stool container. We accepted urine and stool specimens that were delivered within 12 hours of collection. Nine milliliters of blood was collected in tubes with gel serum separators and kept at 4°C. All specimens upon receipt, except blood, were kept at –70°C until processed. Weekly collections of all the specimens were repeated until 42 days after discharge. Information on demographics and clinical course was collected.

### Laboratory Test Methods

Laboratory tests were performed at the Department of Pathology, Singapore General Hospital. Reverse transcription–polymerase chain reaction (RT-PCR) was performed on conjunctival, throat, stool, and urine specimens with the RealArt HPA-coronavirus RT-PCR kit (Artus Gmbh, Hamburg, Germany) on the Roche Lightcycler (Roche Diagnostics Corporation, Indianapolis, IN), a real-time PCR instrument. This assay targeted the polymerase gene of SARS-CoV. All positive results were verified, with two other primer pairs also targeted at the polymerase gene of SARS-CoV. The first were primers designed by the Genome Institute of Singapore (*7*), the second were Cor 1/2 primers from the Government Virus Unit, Hong Kong (sense 5´ CAC CGT TTC TAC AGG TTA GCT AAC GA 3´, antisense 5´ AAA TGT TTA CGC AGG TAA GCG TAA AA 3´) (*8*). Detection limit was set at 1 copy/µL of specimen. Serum specimens were tested for total virus-specific antibodies with an indirect enzyme immunoassay (EIA) with SARS-CoV lysate as the antigen (*9*). Positive serum samples were retested for immunoglobulin (Ig) G by immunofluorescence assay (IFA) with SARS-CoV–infected Vero cells spotted onto microscope slides.

### Statistical Analysis

During recruitment, patients were selected on the basis of the number of days after the patient was discharged from the hospital. During analysis, this date was converted to day of illness, and subsequently week of illness, by determining the date of onset of illness. Onset of illness was defined as the day of onset of fever. For example, specimens collected from day 15 to 21 of illness were thus grouped into week 3 of onset of illness. Statistical analyses were performed with SPSS for Windows release 10.0.1 (SPSS, Inc., Chicago, IL). Categorical variables were analyzed with chi-square test. The student *t* test was used to analyze continuous variables.

## Results

### Patients

**Figure 1:**
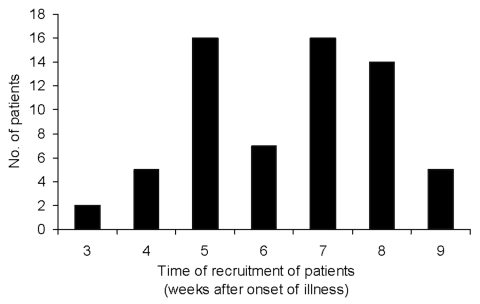
Recruitment of patients by week of illness.

A total of 170 patients met the selection criteria, 64 patients consented to the study, and 4 patients withdrew after the first collection. The mean age of consenting patients was 35.2 years (range 17–63 years), and 25% were men. The mean age of all SARS patients in Singapore was 35 years (range 1.3– 90 years), and 32.4% were male. Patients for this study were recruited from week 3 to 9 of their illness (median week 7) (Figure 1). Most patients (89%) were recruited after week 4 of illness.

Ten participants had coexisting conditions (diabetes mellitus, hypertension, asthma). Four had a history of previous intensive care admission. During their hospital stay, six had history of steroid treatment, defined as prior use of hydrocortisone, prednisolone, dexamethasone, or pulsed methylprednisolone, regardless of dosage and frequency. Ribavirin was used in the treatment of 32 patients (50%).

### Laboratory Investigations

Since an interim analysis on 60 specimens indicated no positive yields 14 days after discharge, a decision was made to stop all subsequent collections until after day 28 of discharge. Specimens already collected were processed.

In total, 126 conjunctival specimens, 124 throat swab specimens, 116 stool specimens, 124 urine specimens, and 123 blood specimens were available for analysis. An average of 9.6 specimens was taken per patient (range 4–19). All patients had at least one specimen of each type taken.

### RT-PCR

**Figure 2:**
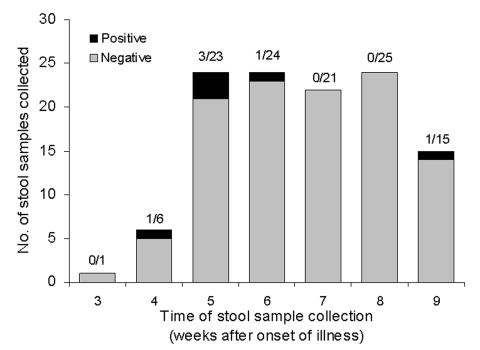
Results of severe acute respiratory syndrome–associated coronavirus polymerase chain reaction (PCR) on stool samples plotted by week of illness. Samples were processed for reverse transcription (RT)-PCR. Any result was deemed positive if it was detected by both the RealArt HPA-coronavirus RT-PCR kit (Artus Gmbh, Hamburg, Germany) and two other RT-PCR primers designed by Genome Institute of Singapore and the Government Virus Unit, Hong Kong.

Most (76%) specimens collected for RT-PCR testing were taken from week 6 to 8 of illness (mean 6.3 weeks, standard deviation [SD] 1.47). All specimens from throat, conjunctiva, and urine were negative for SARS-CoV by RT-PCR. Six stool specimens from five patients were positive by the three RT-PCR assays (Figure 2).

Relevant clinical histories and laboratory data for these patients are summarized in the Table. The five patients with positive viral RNA in stool specimens had a mean age of 31.6 years; two (40%) of the five patients required supplemental oxygen. No patients were admitted to intensive care. Mean peak lactate dehydrogenase (LDH) was 811 U/L. The 59 patients with stool samples negative for SARS-CoV RNA had a mean age of 35.9 years, 33% required supplemental oxygen, and 4 were previously admitted to intensive care. Mean peak LDH for the patients without RNA detected in stool samples was 764 U/L. Age, prior supplemental oxygen use, and peak LDH were not statistically different in the two groups.

**Table:** Clinical and laboratory parameters of patients with stool samples positive for SARS-CoV RNA^a^

Patient no.	Age	Sex	Ethnicity	Occupation	Specimens provided	Viral load^b^ (copies/mL)	History of ICU admission	Required supplemental oxygen	Peak LDH U/L	Concurrent illness
3	43	Male	Malay	Unemployed	Wk 5^c^	Wk 5 ( 33.9 x 10^3^)	No	No	299	Diabetes mellitus type 2 Steroid use
16	33	Female	Indian	Domestic maid	Wks 4,^c^ 5,6	Wk 4 (37.1 x 10^3^)	No	Yes, I/N	1,127	None
35	17	Male	Chinese	Student	Wk 9^c^	Wk 9 (1.73 x 10^3^)	No	No	615	None
51	30	Female	Filipino	Healthcare worker	Wks 5,^c^ 6^c^	Wk 5 (2 x 10^3^) Wk 6 (1.64 x 10^3^)	No	No	1,065	None
62	35	Male	Chinese	Diver	Wk 5^c^	Wk 5 (7.76 x 10^3^)	No	Yes, I/N	952	None

^a^SARS-CoV, severe acute respiratory syndrome–associated coronavirus; ICU, intensive care unit; LDH, lactate dehydrogenase; I/N, intranasal oxygen. ^b^RT-PCR was performed by using the RealArt HPA-coronavirus RT-PCR kit (Artus Gmbh, Hamburg, Germany) on the Roche Lightcycler. Viral load was determined for each mL of stool sample. ^c^Positive RT-PCR results for SARS-CoV.

Patients 3 and 62 provided one sample each and withdrew from the study immediately thereafter. Both were positive for SARS-CoV by RT-PCR in week 5 of illness. No subsequent specimens were available for analysis. Three stool specimens were taken from patient 16 on weeks 4–6 of illness. Only specimens taken in week 4 were positive. Patient 51 provided two stool specimens for week 5 and 6; both were positive. The sample taken in week 6 had fewer copies per reaction on RT-PCR compared to that taken in week 5. A sample requested by her physician, outside this study, was collected on week 10 of illness and tested negative by RT-PCR. Patient 35 provided one sample, which was positive, at week 9 of illness. No subsequent specimens were available.

Of the five patients with positive SARS-CoV detected by RT-PCR in the stool sample, one (patient 3) had a history of prior steroid treatment during SARS illness. As mentioned earlier, this patient provided one sample before withdrawing from the study. Five other patients received steroid treatment during their hospitalization, but none had SARS-CoV detected by RT-PCR. These patients were recruited from week 5 to 7 of illness.

Of the 32 patients who received ribavirin, two had samples positive for SARS-CoV RNA. In comparison, 3 of the 32 patients who did not receive ribavirin had SARS-CoV detected by RT-PCR. This difference was not significant. Diarrhea was reported in 19 (30%) of our study patients; one had a sample positive for SARS-CoV RNA. The remaining RNA-positive samples were from patients who did not report any diarrhea symptoms during their hospital stay. Ten of our participants had a history of chronic illnesses. Three had asthma, five had hypertension, and one had diabetes mellitus type 2. One additional patient had diabetes mellitus type 2 and hypertension. Samples were collected from these patients on weeks 5 to 9 of illness (mean 6.3 weeks [SD 1.22]). None of these patients had SARS-CoV viral RNA detected in our study.

### Serologic Testing

**Figure 3:**
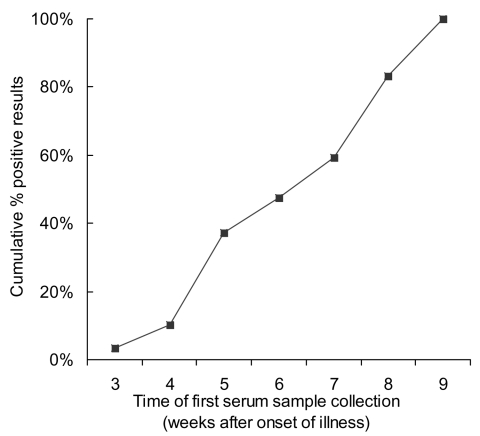
Cumulative results of first serologic testing of samples by week of illness. Serologic testing was performed by indirect enzyme immunoassay with severe acute respiratory syndrome–associated coronavirus lysate as the antigen.

A total of 123 specimens from 64 patients were available for analysis. All specimens were positive by EIA, with the exception of two specimens taken at week 7 and 6 from patients 4 and 5, respectively. All positive results were corroborated with a positive IFA, except for a sample from patient 38, which was negative by IFA on week 5 of illness. The next sample taken on week 6 was positive on both EIA and IFA. The result of the first sample of blood taken for serologic testing was plotted against the respective week of illness (Figure 3).

For patients 5 and 4, whose initial results were negative, serologic test results became positive at the subsequent week of testing (weeks 7 and 8, respectively)

## Discussion

SARS-CoV detected in mucosal secretions or excreta will affect decisions about discharging patients. Detecting SARS-CoV would have a greater importance if it was detected in the respiratory tract (throat swabs) or tears (conjunctival swabs). By testing samples from patients who were discharged from the hospital, we accessed patients deemed clinically fit to return to the community. A positive RT-PCR result in these patients would be more important compared to results of those patients recovering in the hospital. Weekly tests were conducted to assess this risk by using molecular diagnostic methods. This is the first longitudinal cross-sectional study of viral shedding in convalescent-phase SARS patients.

By performing weekly tests, the optimal time of seroconversion in recovering patients could be determined. This information will assist clinicians in planning a reasonable time for serologic testing in patients with suspected cases of SARS.

In our study, viral nucleic acid for SARS-CoV was detected only in the stool samples. This finding was infrequent; most patients (>95%) did not have positive samples after week 5 of illness (Figure 2). One patient still had detectable viral RNA at week 9 of illness. None of our patients had viral nucleic acid for SARS-CoV detected in respiratory, urine, or conjunctival samples.

Detection of viral nucleic acid for SARS-CoV in stool samples of recovering patients has been reported. Samples were positive up to day 25 in a study by Drosten et al. (*3*) and at day 73 in a study by Leung et al. (*10*). The Drosten et al. study involved two patients; both had viral RNA detected during the convalescent phase on day 19 and 25 of illness. In the Leung et al. study, the authors examined the gastrointestinal manifestations of the 138 patients with SARS who were admitted to a local hospital. The overall detection rate of SARS-CoV RNA in stool specimens was 16%. Whether these samples were taken during the acute phase, convalescent phase, or both is unknown. The authors observed that in one patient, viral RNA was detected in a stool sample up to 73 days after symptom onset. No other clinical information was available for this patient.

In a later study, Chan et al. (*11*) reported that viral RNA was detected in ≈5% of stool samples submitted after week 7 of illness. The last positive sample was detected at week 11 of illness; in that study, the respiratory samples (tracheal aspirates, nasopharyngeal aspirates, throat swabs, throat washings, nasal swabs, and pooled throat and nasal swabs) were detected at week 8 of illness. All those who shed virus for a prolonged period (arbitrarily defined as >6 weeks after onset of symptoms) had samples collected while still critically ill.This study had a different cohort of patients compared to ours. Our patients had been discharged and had comparatively shorter and milder illnesses. Determining whether positive carriage persisted in these critically ill patients from the Chan et al. study after discharg would be beneficial.

SARS-CoV was not isolated in this study, so we were unable to assess if those with positive stool samples were infectious. However, in the Chan et al. study (*11*), 2 (1%) of the 195 samples submitted for stool isolation were positive, only during week 1 of illness. In the study by Leung et al. (*10*), virus was not isolated from stool samples. This finding was similar to our own experience in Singapore. Our laboratory was the primary center for SARS-CoV isolation during the epidemic, and we had not been successful in isolating viruses from stool samples. A stool sample positive for SARS-CoV, from convalescent-phase SARS patients, would probably have a minimal effect on the public. Secondary SARS cases from convalescent-phase SARS patients discharged from the hospital have not been reported. We, however, still recommend caution, especially in pediatric patients and adults requiring full nursing aid, since SARS-CoV can survive for at least 2 to 4 days at room temperature (*12*).

With a greater severity of illness, excretion of the virus would likely be prolonged. We identified severity of illness by a history of supplemental oxygen use, admission to intensive care, or a high peak LDH. Peak LDH has been identified along with advanced age as predictors of adverse outcomes in Hong Kong (*13**,**14*). However, in our study patients, age, supplemental oxygen use, and peak LDH were not statistically different between those who had positive stool samples and those who had negative stool samples. Samples from all four patients with a previous history of intensive care admission were negative for SARS-CoV RNA by RT-PCR. This finding suggests that the duration of viral RNA excretion in the convalescent phase may not be solely determined by severity of illness. However, the number of intensive care patients in the study was small.

Six patients (9.4%) in our study received steroid treatment compared to 17.2% of the SARS cohort in Singapore. Stool samples from one patient were positive for RNA. Possible explanations for this finding include the small numbers of those treated with steroids in our study and the fact that all those who had stool samples negative for RNA who were on steroids were recruited late in their illness (weeks 5–7).

SARS-CoV viral RNA was not detected in stool samples from the 10 patients with chronic illnesses. All those who did have SARS-CoV viral RNA had no history of chronic illnesses. Again, a possible explanation is the recruitment of patients late in their illness.

The use of ribavirin did not appear to reduce the detection rates of viral RNA in the stool sample. This finding concurred with observations from in vitro studies (*15*) and retrospective cohort studies (*16*), which suggested that ribavirin was not effective against SARS-CoV. In a separate study, Leong et al. (*17*) showed that ribavirin did not confer any survival benefit for patients with SARS.

Active replication of SARS-CoV has been reported in the small and large intestine (*10*). Diarrhea was not associated with a higher proportion of patients with SARS-CoV–positive stool samples. The increased intestinal motility caused by diarrhea might lessen the viral load in the gastrointestinal tract in those patients with symptoms.

In Hong Kong (*4*), IgG seroconversion was detected from day 10 of illness (mean of 20 days). Ninety-three percent of patients seroconverted by day 30 (week 5) of illness. As part of the protocol, all patients received steroids (intravenous hydrocortisone, oral prednisolone, or pulse methylprednisolone) on diagnosis of SARS. Steroids did not appear to affect seroconversion rates. The Toronto group (*18*) reported similar results. Seropositivity rate was 96.2% at 28 days of illness and beyond.

All but two of our patients demonstrated positive EIA results at the initial specimen collection. These results were expected, since most patients were recruited after week 4 of illness. At week 6 of illness, 27 (96%) of 28 patients had a positive serologic result. Patients 5 and 4 demonstrated seroconversion at week 7 and 8 of illness, respectively. This finding is congruent with the Hong Kong study; therefore, the diagnostic role of serologic testing is limited in the acute phase of illness.

Steroid use was infrequent (6 patients) in our study. Patient 5 received intravenous hydrocortisone during her hospital stay. The other five participants who received steroids during their hospital stay had detectable antibodies at the first specimen collection, performed at week 5 (four patients) and week 7 (one patient). Patient 4 seroconverted at week 8, and she had no prior steroid use, which suggests that the serologic response is not muted by the use of steroids. The two patients who seroconverted during these sequential weekly collections had ribavirin and symptoms of diarrhea during their hospital stay.

Our study had limiting factors. The design of this study was cross-sectional, which did not allow us to accurately determine when seroconversion occurred or when viral RNA ceased to be detectable. Most of our patients did not have severe illness, and most intensive care patients declined to participate. Returning to the hospital on a weekly basis for specimen sampling was also difficult because these patients had substantial sequelae from the infection. Even when recruited, participants were already in the later half of their convalescence. These patients have had more severe illness and would have had a longer hospital stay. At the time of discharge, they would have been beyond week 4 of illness. Our study had few participants in the early phase of recovery. The outbreak in Singapore was already under control when the study commenced at the end of April, which further limited the number of eligible participants in the early convalescent phase.

## Conclusion

Detection of SARS-CoV RNA is uncommon in recovering patients discharged from the hospital. In this study, RT-PCR determined that 5 (7.8%) of 64 patients continued to shed viral RNA in stool samples only, up to week 8 of illness. Most seroconversions (96%) occurred by week 6 of illness, although seroconversion may still occur at week 7 and 8 of illness. Excretion of viral RNA and seroconversion did not appear to be related to age, underlying conditions, diarrhea, prior steroid or ribavirin use, or severity of illness. No secondary cases of infection occurred among the convalescent-phase patients.

## References

[1] World Health Organization. Summary table of SARS cases by country, November 1, 2002–August 7, 2003 [monograph on the Internet]. Geneva: The Organization; 2003 [cited 2003 Aug 20]. Available from http://www.who.int/csr/sars/country/en/country2003_08_15.pdf

[2] Department of Health. HKSAR. Outbreak of severe acute respiratory syndrome (SARS) at Amoy Gardens, Kowloon Bay, Hong Kong [monograph on the Internet]. Hong Kong: Department of Health; 2003 [cited 2003 Jun 2]. Available from http://www.info.gov.hk/info/ap/pdf/amoy_e.pdf

[3] Drosten C, Gunther S, Preiser W, van der Werf S, Brodt HR, Becker S, Identification of a novel coronavirus in patients with severe acute respiratory syndrome. N Engl J Med. 2003;348:1967–76. 10.1056/NEJMoa030747 12690091

[4] Peiris JS, Chu CM, Cheng VC, Chan KS, Hung IF, Poon LL, Clinical progression and viral load in a community outbreak of coronavirus-associated SARS pneumonia: a prospective study. Lancet. 2003;361:1767–72. 10.1016/S0140-6736(03)13412-5 12781535 PMC7112410

[5] World Health Organization. Case definitions for surveillance of severe acute respiratory syndrome (SARS) [monograph on the Internet]. Geneva: The Organization; 2003 [cited 2003 Aug 20]. Available from http://www.who.int/csr/sars/casedefinition/en/

[6] World Health Organization. WHO hospital discharge and follow-up policy for patients who have been diagnosed with severe acute respiratory syndrome (SARS) [monograph on the Internet]. Geneva: The Organization; 2003 [cited 2003 Jun 24]. Available from http://www.who.int/csr/sars/discharge/en/

[7] Ng LF, Wong M, Koh S, Ooi EE, Tang KF, Leong HN, Detection of severe acute respiratory syndrome coronavirus in blood of infected patients. J Clin Microbiol. 2004;42:347–50. 10.1128/JCM.42.1.347-350.2004 14715775 PMC321706

[8] World Health Organization. RT-PCR primers for SARS developed by WHO Network laboratories [monograph on the Internet]. Geneva: The Organization; 2003 [cited 2003 Oct 19]. Available from http://www.who.int/csr/sars/primers/en/

[9] Ksiazek TG, Erdman D, Goldsmith CS, Zaki SR, Peret T, Emery S, A novel coronavirus associated with severe acute respiratory syndrome. N Engl J Med. 2003;348:1953–66. Epub 2003 Apr 10. 10.1056/NEJMoa030781 12690092

[10] Leung WK, To KF, Chan PK, Chan HL. Enteric involvement of severe acute respiratory syndrome-associated coronavirus infection. Gastroenterology. 2003;125:1011–7. 10.1016/j.gastro.2003.08.001 14517783 PMC7126982

[11] Chan PKS, To W-K, Ng K-C, Lam RKY, Ng T-K, Chan RCW, Laboratory diagnosis of SARS. Emerg Infect Dis. 2004;10:825–31. 15200815 10.3201/eid1005.030682 PMC3323215

[12] World Health Organization. First data on stability and resistance of SARS coronavirus compiled by members of WHO laboratory network [monograph on the Internet]. Geneva: The Organization; 2003 [cited 2003 Jun 24]. Available from http://www/who.int/csr/sars/survival_2003_05_04/en/print.html

[13] Lee N, Hui D, Wu A, Chan P, Cameron P, Joynt GM, A major outbreak of severe acute respiratory syndrome in Hong Kong. N Engl J Med. 2003;348:1986–94. 10.1056/NEJMoa030685 12682352

[14] Tsui PT, Kwok ML, Yuen H, Lai ST. Severe acute respiratory syndrome: clinical outcome and prognostic correlates. Emerg Infect Dis. 2003;9:1064–9. 14519241 10.3201/eid0909.030362 PMC3016795

[15] Centers for Disease Control and Prevention. Severe acute respiratory syndrome (SARS) coronavirus testing—United States, 2003. MMWR Morb Mortal Wkly Rep. 2003;52:297–302. 12731699

[16] Booth CM, Matukas LM, Tomlinson GA, Rachlis AR, Rose DB, Dwosh HA, Clinical features and short-term outcomes of 144 patients with SARS in the greater Toronto area. [Erratum in: JAMA. 2003 Jul 16;290:334]. JAMA. 2003;289:2801–9. Epub 2003 May 06. 10.1001/jama.289.21.JOC30885 12734147

[17] Leong H-N, Ang B, Earnest A, Teoh C, Xu W, Leo Y-S. Investigational use of ribavirin in the treatment of severe acute respiratory syndrome, Singapore, 2003. Trop Med Int Health. 2004;9:923–7. 10.1111/j.1365-3156.2004.01281.x 15303999 PMC7169743

[18] Tang P, Louie M, Richardson SE, Smieja M, Simor AE, Jamieson F, Interpretation of diagnostic laboratory tests for severe acute respiratory syndrome: the Toronto experience. CMAJ. 2004;180:47–54. 14707219 PMC305313

